# Treatment of lactating sows with clofibrate as a synthetic agonist of PPARα does not influence milk fat content and gains of litters

**DOI:** 10.1186/s12917-015-0368-y

**Published:** 2015-03-07

**Authors:** Denise K Gessner, Birthe Gröne, Susann Rosenbaum, Erika Most, Sonja Hillen, Sabrina Becker, Georg Erhardt, Gerald Reiner, Klaus Eder

**Affiliations:** Institute of Animal Nutrition and Nutritional Physiology, Justus-Liebig-University Giessen, Heinrich-Buff-Ring 26-32, 35392 Giessen, Germany; Department of Veterinary Clinical Sciences, Swine Diseases, Justus-Liebig-University, Frankfurter Straße 112, 35392 Giessen, Germany; Institute for Animal Breeding and Genetics, Justus-Liebig-University, Ludwigstraße 21b, 35390 Giessen, Germany

## Abstract

**Background:**

In rats, it has been observed that treatment with activators of peroxisome proliferator-activated receptor α (PPARα) disturbs metabolic adaptations during lactation, which in turn lead to a reduction of milk fat content and gains of litters during the suckling period. It has not yet been investigated whether agonists of PPARα are impairing milk production of lactating sows in a similar manner as in rats. Therefore, the present study aimed to investigate the effect of treatment with clofibrate, a strong synthetic agonist of PPARα, on milk composition and litter gains in lactating sows.

**Results:**

Twenty lactating sows received either a basal diet (control group) or the same diet with supplementation of 2 g of clofibrate per kg of diet (clofibrate group). In the clofibrate group, mRNA concentrations of various PPARα target genes involved in fatty acid utilization in liver and skeletal muscle were moderately up-regulated. Fat and energy content of the milk and gains of litters during the suckling period were not different between the control group and the clofibrate group.

**Conclusion:**

It is shown that treatment with clofibrate induces only a moderate up-regulation of PPARα target genes in liver and muscle of lactating sows and in turn might have limited effect on whole body fatty acid utilization. This may be the reason why clofibrate treatment did not influence milk fat content and gains of litters during the suckling period. Thus, the present study indicates that activation of PPARα induced either by native agonists such as dietary polyunsaturated fatty acids or a by negative energy balance might be largely uncritical in lactating sows with respect to milk production and litter gains in lactating sows.

## Background

Lactation is a physiological state which in most animal species is associated with a strong increase in energy and nutrient requirement for milk production. This demand is usually met by marked increase in feed intake and by utilization of body’s energy stores. In some animal species such as rats or mice, additionally metabolic adaptations occur during lactation which aim to conserve energy for milk production [1-3]. Down-regulation of proteins involved in fatty acid uptake and oxidation in liver and skeletal muscle and a decreased thermogenesis contribute to these energy sparing effects [3-6]. We have observed that these effects in lactating rodents are mediated by a suppression of peroxisome proliferator-activated receptor α (PPARα), a transcription factor which controls the expression of many genes involved in lipid catabolism [7,8]. In lactating rats, it has been moreover observed that an activation of PPARα by feeding an oxidized fat causes a disturbance of those metabolic adaptations. It has been found that there was an increased uptake of fatty acids into the liver and an increased oxidation of fatty acids in the lactating rats fed the oxidized fat, which in turn led to a reduced availability of fatty acids for milk production, resulting in a reduced milk triacylglycerol (TAG) concentration and reduced growth rates of the suckling pups [9].

In lactating sows, milk production is important for the post-natal growth of their litters. However, in contrast to rodents, the role of PPARα in lactating sows has not yet been determined. According to rodent studies, an activation of PPARα during lactation could suppress milk fat synthesis due to a reduced availability of fatty acids as a result of an increased utilization of fatty acids by liver and muscle. The question whether activation of PPARα could affect milk fat synthesis is of relevance as dietary components such as polyunsaturated fatty acids (PUFA) are able to activate PPARα [10-12]. Moreover, it is known that a negative energy balance which is common in lactating sows causes an activation of PPARα due to increased concentrations of NEFA in plasma released from adipose tissue [13-15]. Indeed, recently an activation of PPARα in liver of lactating sows with a negative energy balance has been observed [16-18]. However, metabolic effects of activation of PPARα in lactating sows have not been investigated so far. In order to investigate the hypothesis that activation of PPARα could impair milk production, we performed an experiment with lactating sows which were fed a diet supplemented with clofibrate as a synthetic agonist of PPARα and determined milk composition and gains of litters.

## Methods

### Animals and diets

Twenty second parity sows (Large White and German Landrace) were artificially inseminated and kept in single crates until day 21 of pregnancy. From day 21 to 110 of pregnancy, the sows were kept in groups in pens equipped with fully slatted floors, nipple drinkers and feeders with a temperature of 19 ± 1°C with a 12:12 h light–dark cycle and 60–80% relative humidity by means of an air conditioning system. On day 110 of pregnancy, they were housed individually in farrowing crates. After parturition, the sows were randomly assigned into two feeding groups of 10 animals each with an equalized litter size of 8 piglets per sow. Infrared heaters were used to provide a surrounding temperature of 35°C for newborn pigs. The duration of the suckling period was 21 days.

During lactation all sows were fed a nutritionally adequate basal diet consisting mainly of (in g/kg diet): wheat (325), barley (400), soybean meal with 43% crude protein (190), a mixture of soybean oil and palm oil (50) (1:4, w/w) and a mineral supplement (35) (Sauengold Lac®, Sano-Moderne Tierernährung GmbH, Loiching, Germany). The basal diet contained 13.8 MJ metabolizable energy (ME)/kg; concentrations of crude nutrients were (g/kg diet): Crude protein, 154; crude fat, 74; crude fiber, 45; crude ash, 43; starch, 395. Major fatty acids of the basal diet were (g/100 g of total fatty acids): Palmitic acid (16:0), 31.5; stearic acid (18:0), 3.9; oleic acid (18:1), 30.7; linoleic acid (18:2 n-6), 29.4; α-linolenic acid (18:3 n-3) 2.0. The control group received the basal diet without any further supplement. The second group (“clofibrate group”) received the basal diet supplemented with 2 g of clofibrate (Sigma-Aldrich, Schnelldorf, Germany) per kg of diet. At the day of parturition, the sows were given 3 kg of the diet. From day 2 of lactation to later lactation, the amount of diet administered was calculated in order to meet the energy requirement of the sows (assuming daily litter gains of 2 kg) [19]. Water was given *ad libitum* by nipple drinkers. Feed intakes were recorded daily and body weights weekly. The energy balance of the sows during lactation was estimated as the difference of ME intake and total energy requirement for maintenance and energy requirement for milk production. It was assumed that milk production for 1 kg of litter gain is equivalent to an energy demand of 29.3 MJ ME [19]. The piglets were offered a creep feed (Bonni M-Forte, Sano-Moderne Tierernährung) from day 15 until weaning.

All experimental procedures were carried out in accordance with established guidelines for the care and handling of laboratory animals and were approved by the local Animal Welfare Authorities (Regierungspräsidium Giessen; permission no: GI 19/3-No. 29/2010).

### Sample collection

On day 20 of lactation, blood from *Vena jugularis* was collected 3 h after feed intake into heparinized polyethylene tubes (Sarstedt, Nürnberg, Germany) and plasma was subsequently obtained by centrifugation of the blood (1100 × g, 10 min, 4°C) and stored at −20°C. On day 20, liver and muscle biopsy samples were taken percutaneously after anaesthesia by intramuscular injection of 2 mg azaperon (Stresnil, Janssen, Germany) per kg body mass, 20 mg ketamine (Ursotamin, Serumwerke Bernburg AG, Germany) per kg body mass and up to 2.4 mg thiopental (Thiopental 0.5 g, Inresa, Freiburg, Germany) per kg body mass as required for maintenance of anaesthesia. The pigs were placed in the left lateral recumbency and the surgical area was infiltrated with 1 mL of 2% lidocaine hydrochloride (BelaPharm, Vechta, Germany) for local anaesthesia. A percutaneous liver biopsy was performed with a 16 G/1.65 mm biopsy needle (length: 160 mm) on a HistoCore® system (BIP Biomed. Instrumente & Produkte GmbH, Türkenfeld, Germany) on expected anatomical location of the liver under constant ultrasound guidance. The intramuscular biopsy (*M. longissimus dorsi)* was performed with a spirotome (8 G, Medinvents, Hasselt, Belgium). After removal of the samples, the sites for biopsy were subsequently closed with synthetic absorbable surgical suture. For collection of milk samples, sows were given 20 I.U. oxytocin (CP-Pharma, Burgdorf, Germany) by intramuscular injection. 80–100 ml of milk was expressed manually from all active teats of each sow. Liver and muscle samples were stored at −80°C and milk samples were stored at −20°C pending analysis.

### Concentrations of nutrients in milk

Measurements of protein and fat concentrations in the milk were performed by the official methods of Verband Deutscher Landwirtschaftlicher Untersuchungs- und Forschungsanstalten (VDLUFA) [20]. The concentration of lactose in the milk was determined using a commercial kit (cat. no. 10986119035, R-Biopharm, Darmstadt, Germany) according to the manufacturer’s instructions. The gross energy content of the milk was calculated using the following gross energy values: fat = 39 kJ/g, protein = 24 kJ/g, lactose = 17 kJ/g. For analysis of the fatty acid composition of milk total lipids, lipids were extracted from the milk with a hexane:isopropanol (3:2, v/v) mixture [21]. Fatty acids were analyzed by capillary gas chromatography after converting fatty acids into their methyl esters by trimethyl sulfonium hydroxide [22]. Concentrations of retinol and α-tocopherol in milk samples were determined by HPLC (L-7100, LaChrom, Merck-Hitachi, Darmstadt, Germany) using a modification of the method of Balz *et al.* [23]. Samples of 0.5 ml of milk were mixed with 2 ml of a 10 g/l pyrogallol solution (in ethanol, absolute) and 300 μl of a saturated sodium hydroxide solution. After flushing with nitrogen, this mixture was heated for 30 min at 70°C in closed glass tubes. Retinol and α-tocopherol were then extracted by addition of 2 ml of n-hexane and 2 ml of bidest. water. After centrifugation, an aliquot of the hexane phase was evaporated to dryness under nitrogen and re-dissolved in methanol containing 0.05% of butylated hydroxytoluene. Retinol and α-tocopherol were separated isocratically by HPLC using a mixture of methanol and water (96:4, v/v) as mobile phase and a LiChrosher 100 RP18 column (5 μm particle size, 125 mm length, 4.6 mm internal diameter, Merck, Darmstadt, Germany) and detected by fluorescence (Fluorescence Detector L-7480, LaChrom, Merck-Hitachi, Darmstadt, Germany; retinol: excitation wavelength, 325 nm, emission wavelength, 475 nm; α-tocopherol: excitation wavelength, 295 nm, emission wavelength, 325 nm).

### Concentration of TAG and NEFA in plasma

Plasma concentrations of TAG and NEFA were measured using enzymatic kits [Fluitest® TG, Cat. No. 5741, Analyticon® Biotechnologies AG, Lichtenfels, Germany; NEFA-HR (2), Code No. 436–91995, Wako Chemicals GmbH, Neuss, Germany].

### RNA isolation and qPCR

RNA isolation from frozen liver and muscle biopsies and quantitative real-time PCR (qPCR) analysis were performed as described recently in detail [24]. In brief, total RNA from frozen liver and muscle samples was isolated using Trizol® Reagent (Invitrogen, Karlsruhe, Germany) according to the manufacturer’s protocol and purified using the RNeasy Minikit (Qiagen, Hilden, Germany). Concentration and purity of the RNA were estimated from the optical density at 260 and 280 nm, respectively. The integrity of total RNA was verified using 1.2% agarose gel electrophoresis. cDNA was synthesized using 1.2 μg of total RNA, 100 pmol oligo(dT)_18_ primer (Eurofins MWG Operon, Ebersberg, Germany), 1.25 μl 10 mM dNTP mix (GeneCraft, Lüdinghausen, Germany), 5 μl 5x RT reaction buffer (Thermo Fisher Scientific, St. Leon-Rot, Deutschland), and 60 units M-MuLV Reverse Transcriptase (Thermo Fisher Scientific) at 42°C for 60 min and a final inactivating step at 70°C for 10 min in a thermo cycler (Biometra, Göttingen, Germany). qPCR runs were performed with a Rotorgene 2000 system (Corbett Research, Mortlake, Australia) using KAPA SYBR FAST qPCR Universal Mastermix (Peqlab, Erlangen, Germany) and gene-specific primer pairs from Eurofins MWG Operon (Ebersberg, Germany). Ct-values of target and reference genes were obtained using Rotorgene Software 5.0. Determination of relative expression levels was calculated using GeNorm normalization factor including the three most stable out of six reference genes [25]. Gene-specific primer pairs were designed intron-spanning if possible using Primer3 and BLAST. Characteristics of primers used for qPCR analysis are shown in Table 1.

**Table 1:** **Characteristics of gene specific primers used for qPCR**

**Gene**	**Forward primer (from 5′ to 3′)**	**PCR product size (bp)**	**NCBI GenBank**	**Slope**	**R** ^**2**^	**Efficiency**	**M-value**
	**Reverse primer (from 5′ to 3′)**						
*Reference genes*							
*ACTB*	GACATCCGCAAGGACCTCTA	205	XM_003124280.3	−3.76	1.000	0.84	0.034
	ACATCTGCTGGAAGGTGGAC						
*ATP5G1*	CAGTCACCTTGAGCCGGGCGA	94	NM_001025218.2	−3.43	0.999	0.99	0.033
	TAGCGCCCCGGTGGTTTGC						
*GSR*	AGCGCGATGCCTACGTGAGC	175	XM_003483635.2	−3.43	0.997	0.96	0.038
	GGTACGCCGCCTGTGGCAAT						
*RPS9*	GTCGCAAGACTTATGTGACC	325	XM_005664825.1	−3.78	0.999	0.84	0.034
	AGCTTGAAGACCTGGGTCTG						
*PPAR*α *target genes*							
*ACOX1*	CTCGCAGACCCAGATGAAAT	218	NM_001101028.1	−3.53	0.999	0.92	
	TCCAAGCCTCGAAGATGAGT						
*CD36*	TCCTCTGACATTTGCAGGTCAATCT	107	NM_001044622.1	−3.59	0.999	0.90	
	GGAGATGCAAAAGCTGTGGATGG						
*CPT1A*	GCATTTGTCCCATCTTTCGT	198	NM_001129805.1	−3.46	0.999	0.95	
	GCACTGGTCCTTCTGGGATA						
*CPT1B*	CACACTGCAGCACCTCACA	183	NM_001007191.1	−4.09	1.000	0.76	
	GTGAGGGCTGCCAGCTTTT						
*CYP4A24*	GGTTTGCTCCTGTTGAATGG	121	NM_214424.1	−3.34	1.000	0.99	
	GCATCACTTGGACAGACTTG						
*FABP1*	ATCGTGCAGAATGGGAAGCA	133	NM_001004046.1	−3.43	0.999	0.96	
	ACTGAACCACTGTCTTGACC						
*FABP3*	CAACATGACCAAGCCTACCA	227	NM_001099931.1	−3.36	1.000	0.98	
	CTAGTTCCCGAACAAGCGTT						
*LPL*	TTCTCCCGACGACGCAGATTTT	166	NM_214286.1	−3.36	0.988	0.98	
	TGCAATCACACGGATGGCTTCT						
*SLC27A1*	GGTTCCAGCCTGTTGAATGT	275	NM_001083931.1	−3.38	0.992	0.98	
	AACAAAACCTTGGTGCTTGG						
*UCP3*	GCCACTTTGTCTCTGCCTTC	219	NM_214049.1	−3.39	1.000	0.97	
	CAAACATCACCACGTTCCAG						
*Genes of the ubiquitin proteasome system*							
*FBXO32*	TCACAGCTCACATCCCTGAG	167	NM_001044588.1	−3.30	0.994	1.01	
	GACTTGCCGACTCTCTGGAC						
*TRIM63*	ATGGAGAACCTGGAGAAGCA	219	NM_001184756.1	−3.62	0.998	0.89	
	ACGGTCCATGATCACCTCAT						
*UBB*	GGTGGCTGCTAATTCTCCAG	127	NM_001105309.1	−3.51	1.000	0.93	
	TTTTGGACAGGTTCAGCTATTAC						

*Abbreviations*: *ACOX1* acyl-CoA oxidase, *ACTB*  actin, beta, *ATP5G1* ATP synthase, H+ transporting, mitochondrial Fo complex, subunit C1, *CD36* fatty acid translocase, *CPT1* carnitine-palmitoyl-transferase, *CYP4A24* cytochrome P450 A24, *FABP* fatty acid binding protein, *FBXO32* F-box protein 32, *LPL* lipoprotein lipase, *GSR* glutathione reductase, *PPAR*α peroxisome proliferator- activated receptor α, *RPS9* ribosomal protein S9, *SLC27A1* solute carrier family 27 (fatty acid transporter), member 1, *TRIM63* tripartite motif containing 63, E3 ubiquitin protein ligase, *UBB* ubiquitin B, *UCP3* uncoupling protein 3.

### Statistical analysis

Statistical analysis was performed by student’s t test using the Minitab Statistical Software Rel. 13.0 (Minitab, State College, PA, USA). Differences were considered statistically significant for *P* < 0.05.

## Results

### Daily feed intake, body weights and energy balance of the lactating sows

The daily feed intake during the three weeks of lactation was not different between control sows and sows administered clofibrate (Table 2). Body weights of the sows at day 1 post partum and day 21 post partum and body weight losses of the sows from day 1 to day 21 were similar in both groups of sows (Table 2). The calculated energy balance of the sows from day 1 post partum to day 14 post partum, the time period in which piglets did not receive any additional feed, was also similar in both groups of sows (Table 2).

**Table 2:** **Daily feed intake, body weights and energy balance of lactating control sows and sows administered clofibrate**

	**Control**	**Clofibrate**	***P*** **-value**
Daily feed intake (kg/d)			
Week 1	6.06 ± 0.65	5.47 ± 0.75	0.088
Week 2	5.88 ± 0.91	6.26 ± 0.63	0.333
Week 3	6.29 ± 0.57	6.28 ± 0.97	0.978
Weeks 1–3	6.08 ± 0.54	6.00 ± 0.67	0.807
Body weight (kg)			
Day 1 p.p.	264 ± 15.4	263 ± 18.3	0.910
Day 21 p.p.	247 ± 21.0	242 ± 27.9	0.688
Body weight loss (kg)			
Day 1 p.p. – day 21 p.p.	16.9 ± 9.14	20.9 ± 16.42	0.550
Energy balance (MJ ME/d),			
Day 1 p.p. – day 14 p.p.	−4.91 ± 13.25	−9.24 ± 8.88	0.436

Values are mean ± SD (n = 10/group); p.p. = post partum.

### mRNA concentrations of PPARα target genes in tissues of the lactating sows

In overall, administration of clofibrate had only a moderate effect on the expression of the PPARα target genes in liver and muscle of sows on day 20 of lactation (Table 3). In the liver, mRNA concentrations of only 2 out of the 7 PPARα target genes considered (*CPT1A* and *CYP4A24*) were significantly up-regulated by administration of clofibrate (*P* < 0.05, Table 3). The extent of up-regulation for these genes, with the only exception of *CPT1A* was moderate. In muscle, there was a significant (*P* < 0.05) up-regulation of 5 out of the 8 PPARα target genes considered (*ACO, CD36, CPT1B, CYP4A24, LPL*). As in the liver, the extent of up-regulation of these genes, being in the range between 43% and 68%, however, was relatively moderate (Table 3).

**Table 3:** **Relative mRNA concentrations of PPAR**α **target genes in liver and muscle of lactating control sows and sows administered clofibrate on day 20 of lactation**

	**Control**	**Clofibrate**	***P*** **-value**
Liver			
*ACOX1*	1.00 ± 0.61	1.16 ± 0.65	0.577
*CD36*	1.00 ± 0.38	0.91 ± 0.39	0.656
*CPT1A*	1.00 ± 0.75	3.86 ± 1.77	0.003
*CYP4A24*	1.00 ± 0.47	1.66 ± 0.57	0.034
*FABP1*	1.00 ± 0.36	1.32 ± 0.42	0.144
*LPL*	1.00 ± 0.49	1.48 ± 0.48	0.104
*SLC27A1*	1.00 ± 0.47	0.96 ± 0.14	0.835
Muscle			
*ACO*	1.00 ± 0.36	1.62 ± 0.38	0.002
*CD36*	1.00 ± 0.16	1.43 ± 0.16	< 0.001
*CPT1B*	1.00 ± 0.37	1.64 ± 0.53	0.001
*CYP4A24*	1.00 ± 0.38	1.52 ± 0.31	0.012
*FABP3*	1.00 ± 0.24	1.27 ± 0.35	0.094
*LPL*	1.00 ± 0.51	1.68 ± 0.48	0.010
*SLC27A1*	1.00 ± 0.10	1.12 ± 0.14	0.101
*UCP3*	1.00 ± 0.32	1.16 ± 0.20	0.296

Values are mean ± SD (n = 10/group); Abbreviations: see Footnote of Table 1.

### Concentrations of TAG and NEFA in plasma

The concentrations of TAG and NEFA in plasma on day 20 of lactation were significantly lower in the clofibrate group than in the control group (*P* < 0.05, Table 4).

**Table 4:** **Concentrations of TAG and NEFA in plasma of lactating control sows and sows administered clofibrate on day 20 of lactation**

	**Control**	**Clofibrate**	***P-*** **value**
TAG (mmol/L)	0.37 ± 0.13	0.23 ± 0.09	0.029
NEFA (μmol/L)	152 ± 66	64 ± 18	0.002

Values are mean ± SD (n = 10/group).

### Milk composition of the lactating sows and gains of litters

Concentrations of fat, lactose and the calculated gross energy content of the milk on day 20 of lactation did not differ between control sows and sows administered clofibrate. However, the concentration of protein in the milk was higher in the clofibrate group than in the control group (Table 5). The fatty acid composition of milk total lipids was slightly different between the two groups of sows. In the milk of sows administered clofibrate, the proportion of palmitic acid (16:0) was slightly higher and proportions of linoleic acid (18:2 n-6) and α-linoleic acid (18:3 n-3) were slightly lower than in milk of control sows (Table 5). Proportions of all the other fatty acids in milk total lipids were unchanged between the two groups of sows (Table 5). According to the differences in the proportions of palmitic acid, linoleic acid and α-linoleic acid, milk of sows administered clofibrate had a higher proportion of total saturated fatty acids and a lower proportion of total polyunsaturated fatty acids (PUFA) than milk of control sows (Table 5). The concentrations of the fat soluble vitamins retinol and α-tocopherol in the milk, expressed per g of fat, were also similar in both groups of sows (Table 5). Gains of litters during the first 14 days (the period in which piglets received sows milk exclusively) and the whole 21 days suckling period were also not different between control sows and sows supplemented with clofibrate (Table 6).

**Table 5:** **Composition of milk of lactating control sows and sows administered clofibrate on day 20 of lactation**

	**Control**	**Clofibrate**	***P-*** **value**
*Nutrients (%)*			
Fat	6.81 ± 0.49	6.92 ± 1.15	0.797
Lactose	4.85 ± 0.47	5.04 ± 0.42	0.367
Protein	5.19 ± 0.39	5.66 ± 0.33	0.012
Gross energy (MJ/kg)^#^	4.73 ± 0.22	4.91 ± 0.47	0.297
*Fatty acids (% of total fatty acids)*			
14:0	4.5 ± 0.7	4.6 ± 0.6	0.856
16:0	27.1 ± 1.6	30.1 ± 2.0	0.006
16:1 n-9	12.4 ± 1.6	12.1 ± 1.0	0.658
18:0	2.9 ± 0.4	3.0 ± 0.4	0.360
18:1 n-9	31.7 ± 3.5	30.8 ± 3.1	0.570
18:1 n-7	1.9 ± 0.3	1.9 ± 0.2	0.819
18:2 n-6	15.2 ± 0.4	13.7 ± 0.7	< 0.001
18:3 n-3	1.2 ± 0.1	1.0 ± 0.1	0.018
Σ SFA	35.3 ± 2.1	38.5 ± 2.7	0.021
Σ MUFA	46.6 ± 2.6	45.3 ± 2.6	0.313
Σ PUFA	18.1 ± 0.7	16.3 ± 0.9	< 0.001
*Fat soluble vitamins (nmol/g fat)*			
Retinol	16.1 ± 3.74	17.9 ± 4.46	0.374
α-tocopherol	83.4 ± 27.1	95.9 ± 24.1	0.314

Values are mean ± SD (n = 10/group); *Abbreviations*: *SFA* saturated fatty acids, *MUFA* monounsaturated fatty acids, *PUFA* polyunsaturated fatty acids. ^#^calculated value.

**Table 6:** **Weights of litters from lactating control sows and sows administered clofibrate at birth and gains of litters during the period from day 1 to day 21 of lactation**

	**Control**	**Clofibrate**	***P*** **-value**
Weights of litters (kg)			
Day 1	12.5 ± 1.28	12.8 ± 1.58	0.657
Day 21	57.4 ± 6.09	58.9 ± 5.22	0.602
Gains of litters (kg)			
Day 1 – day 14	28.0 ± 4.09	29.5 ± 3.96	0.448
Day 1 – day 21	45.0 ± 6.88	46.1 ± 5.25	0.704

Values are mean ± SD (n = 10/group). Litters were standardized to 8 piglets per sow.

### Relative mRNA concentrations of components of the ubiquitin proteasome system in muscle

In order to find out whether an enhanced proteolysis in muscle could be the reason for an increase of milk protein content found in sows treated with clofibrate, mRNA concentrations of *UBB* (encoding ubiquitin), *FBOX32* (encoding atrogin-1) and *TRIM63* (encoding muscle specific RING Finger protein 1, MuRF-1) in muscle were determined. Relative mRNA concentrations of these three genes were higher in sows treated with clofibrate than in control sows [control group vs. clofibrate group: *UBB*, 1.00 ± 0.23 vs. 1.28 ± 0.12 (*P* = 0.009); *FBOX32*, 1.00 ± 0.64 vs. 1.98 ± 0.80 (*P* = 0.025); *TRIM63*, 1.00 ± 0.45 vs. 2.71 ± 0.97 (*P* = 0.001); means ± SD for all genes].

## Discussion

This study was performed to investigate the effect of activation of PPARα on the expression of genes involved in the utilization of fatty acids in liver and muscle of lactating sows. Moreover, we aimed to find out whether alterations in fatty acid utilization could affect milk fat synthesis. To induce activation of PPARα, lactating sows were fed a diet supplemented with clofibrate, a well-known synthetic agonist of PPARα belonging to the group of fibrates [26]. We found that sows fed the diet supplemented with clofibrate indeed showed an up-regulation of several classical PPARα target genes involved in fatty acid uptake and oxidation in liver and muscle which in its extent, however, was weaker than in previous studies with young pigs treated with clofibrate [15,27,28]. The changes in mRNA concentrations of PPARα target genes indicate that the clofibrate treatment of the sows caused a moderate activation of PPARα in liver and muscle. The main reason for the relatively weak up-regulation of PPARα target genes might be that the dose of clofibrate given to the sows in this study (2 g/kg diet, equivalent to 45 mg/kg body weight) was lower than the doses given to young pigs in the recent studies (5 g/kg diet, equivalent to doses of 300–500 mg/kg body weight) [15,27,28]. Moreover, it should be noted that pigs have generally a relatively low expression of PPARα in comparison to some other species such as rodents and target genes are less responsive against agonists [15,27,29]. It has been shown in sows that a negative energy balance commonly observed during lactation could induce activation of PPARα due to increased concentrations of NEFA released from adipose tissue [16,17]. As the control sows considered in this study had only a slight negative energy balance which was reflected by low plasma NEFA concentrations, it is unlikely that there was a pronounced activation of PPARα in tissues of the control sows. Moreover, it is unlikely that there was an activation of PPARα by dietary fatty acids as the dietary fat, a mixture of soybean oil and palmoil (1:4, w/w), provided low amounts of highly unsaturated fatty acids with a high affinity to PPARα.

The finding that plasma TAG and NEFA concentrations were reduced by clofibrate treatment suggests that the moderate up-regulation of PPARα target genes led to an increased utilization of TAG and fatty acids in tissues. Reduced plasma TAG concentrations which have been reported as a typical feature of activation of PPARα in rats and pigs are mainly due to a reduced secretion of lipid from the liver into the blood and an increased clearance of plasma TAG by lipoprotein lipase (LPL) [30,31]. As sows treated with clofibrate showed an up-regulation of LPL in muscle, it is likely that the reduction of plasma TAG concentration was at least in part due to an increased lipolysis. A reduction of plasma NEFA concentration which has also been reported in humans or animals treated with fibrates [32-35] might be mainly due to the up-regulation of fatty acid transporters which are direct targets of PPARα [14]. In the present study, we observed an up-regulation of CD36, one of the key fatty acid transporters, in muscle of sows treated with clofibrate which might contribute to the reduction of plasma NEFA concentration observed in the sows treated with clofibrate.

The finding that fat and energy content of the milk and weight gains of litters during the suckling period (including the first 14 days of suckling in which sows milk was the only source of nutrients) were not different between the two groups of sows shows that the moderate up-regulation of PPARα target genes in liver and muscle by clofibrate did not cause an impairment of milk synthesis in the mammary gland. Sows’ milk fat consists mainly of two sources of fatty acids, namely those deriving from *de-novo* fatty acid synthesis in the mammary gland and those released from TAG-rich lipoproteins by LPL and taken up into the mammary gland. In contrast, plasma NEFA contribute less to the production of milk TAG as the uptake of NEFA into the mammary gland is relatively low in sows in comparison to other species such as cattle [36]. Fatty acids deriving from *de-novo* synthesis are mainly saturated and monounsaturated, while those transported via chylomicrons to the mammary gland are reflecting the dietary fatty acid profile [36]. A reduced plasma TAG concentration in sows treated with clofibrate indicates that there was a lower availability of fatty acids from TAG-rich lipoproteins in the mammary gland which might provide an explanation for the finding that milk of these sows had slightly lower concentrations of PUFA. Nevertheless, this study shows that a moderate reduction of plasma TAG concentration by administration of a strong PPARα agonist does not impair milk fat synthesis.

An unexpected finding of this study was that sows treated with clofibrate showed an increased protein content in the milk. As milk protein synthesis depends on the availability of amino acids in the mammary gland [36], an increased flux of amino acids via blood to the mammary gland could provide a potential explanation for this observation. Recently, we have observed that treatment of rodents with clofibrate stimulates the ubiquitin proteasome system and enhances protein breakdown in muscle [37]. In the present study we observed that treatment of sows with clofibrate causes an up-regulation of ubiquitin and the two E3 ligases atrogin-1 and MuRF-1 which are-rate limiting for the ubiquitin proteasome system. This finding suggests, although we do not have direct evidence for this, that treatment with clofibrate stimulates protein breakdown in muscle of sows which in turn could increase the availability of amino acids for milk protein synthesis. However, this remains a matter of speculation. We moreover observed that concentrations of retinol and α-tocopherol in the milk were not different between the two groups indicating that clofibrate did not influence the metabolism of fat soluble vitamins, i.e. their delivery from TAG-rich lipoproteins into the mammary gland.

It has been established that dietary fatty acids such as n-3 PUFA can act as agonists of PPARα [10-12]. Based on the fact that the PPARα activating potential of those fatty acids is lower than that of synthetic PPARα agonists such as clofibrate, the findings of the present study clearly indicate that fatty acids in sow diets acting as native PPARα agonists might be uncritical with respect to milk production and gains of litters of lactating sows. The study moreover suggests that a moderate up-regulation of PPARα target genes involved in fatty acid uptake and oxidation in liver and muscle as a consequence of a negative energy balance might not interfere with milk production of sows. This suggestion is supported by the observation that lactating sows are able to maintain their milk production even under the condition of a negative energy balance as long as their body fat reserves are not depleted to a large extent [38].

## Conclusions

In overall, the present study shows that treatment of lactating sows with clofibrate, a strong synthetic PPARα agonist, induces only a moderate activation of PPARα in liver and muscle and in turn had limited effect on whole body fatty acid utilization. This may be the reason why clofibrate treatment did not influence milk composition and gains of litters during the suckling period. Thus, the present study indicates that activation of PPARα induced either by native agonists such as dietary PUFA or a by negative energy balance might be largely uncritical in lactating sows with respect to milk production and litter gains in lactating sows.
